# Online Sensor Fault Detection Using Machine Learning Algorithms on a Laboratory-Scale Batch Reactor: LSTM Approach

**DOI:** 10.1021/acsomega.5c11180

**Published:** 2026-02-12

**Authors:** Natasha Chrissane Lobo, Himani H. Poojary, Lubna Katapady, Prerana Rao Adyapady, Arockiaraj Simiyon, Thirunavukkarasu Indiran

**Affiliations:** † Department of Computer Science Engineering, 388888 Shri Madhwa Vadiraja Institute of Technology and Management, Bantakal 574115, India; ‡ Manipal School of Information Sciences, 76793 Manipal Academy of Higher Education, Manipal 576104, India; § Manipal Institute of Technology, Manipal Academy of Higher Education, Manipal 576104, India

## Abstract

This article presents an online fault detection system for a laboratory-scale batch reactor (BR) using the Convolutional Neural Network (CNN)-Squeeze and Excitation-based Improved Multi-Layer Long Short-Term Memory (CS-IMLSTM) model. To identify superimposed and sparse sensor faults in real time, the system continuously monitors the BR parameters, such as reactor temperature, coolant flow rate, and heater current. To reduce noise and dynamic fluctuations, the CS-IMLSTM integrates a channel–spatial attention mechanism and enhances feature significance. The performance of the proposed model is compared with LSTM and CNN-LSTM models. The results indicate that the CS-IMLSTM demonstrates improved accuracy, faster adaptation of online learning, and effectiveness in identifying abnormal circumstances compared with LSTM and CNN-LSTM models. The proposed approach can be used for intelligent predictive maintenance in dynamic industrial environments to enhance the reliability and safety of chemical process operations.

## Graphical abstract

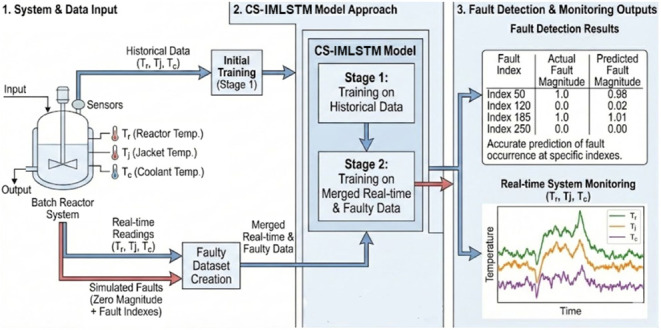

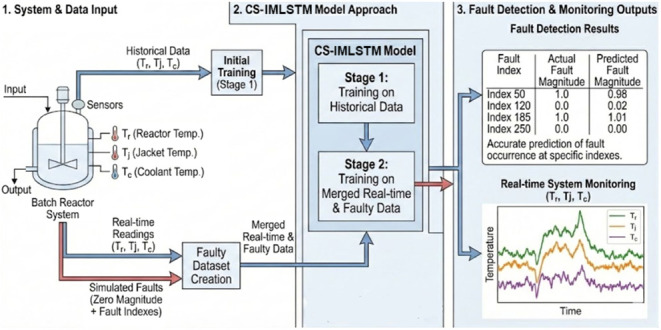

## 1 Introduction

Batch reactors (BRs) are multipurpose, controlled devices used in the chemical and pharmaceutical industries. The BRs also found applications in many other mixing processes, due to their precise control of reactor temperature and pressure from small-scale to medium-scale industries for such multiproduct preparations. BRs provide flexibility and quality control for complex nonlinear operations. Fault detections and diagnosis (FDD) are difficult in BR systems due to their nonlinear dynamics, significant variable coupling, fast-changing operating phases, and nonstationary nature of sensors. Isolating failed sensors and other hardware redundancies is crucial in sensor fault detection and isolation (FDI) methods.^1^

Early FDD research focused on quantitative system-based methods such as heat release models, parameter estimation, and system equations to identify the behaviors of reactor systems.^2^ These models considered basic dynamics of the system. Unexpected disturbances in modeling and parameter uncertainty in dynamic batch processes exist in the systems, showing that they offered only theoretical foundations. Later research proposed multivariate statistical process monitoring methods like Principal Component Analysis (PCA) and other high-dimensional monitoring approaches. To study the residual behavior of the system for anomaly detection, PCA was used to convert sensor readings into uncorrelated latent variables.^3^ Even though PCA was found to be scalable for monitoring, when sensor drift and other related channel failures occur, it could not capture long-range temporal correlations and nonlinear fault sequences. The digital sensing system in the current reactor system changed the direction of research in FDD from the predefined physical assumptions and from the data-driven approaches used to learn directly from multivariate time-series data.

The channel relevance between sensors is modeled to improve the interpretability between them for multivariate spatial dependencies. Techniques like Squeeze and Excitation (SE) for recalibration of features based on attention mechanisms are important adaptive spatial feature weighting methods among different data-driven approaches.^4^ Simultaneously, Long Short-Term Memory (LSTM) networks provide a solid basis for simulating long-term temporal correlations via gating mechanisms and memory cells that preserve crucial fault signals throughout reactor stages.^5^ CNN-LSTM approaches were used to enhance the fault extraction for dynamic chemical process monitoring. This capacity for batch-wise monitoring was further expanded by encoder–decoder based LSTM frameworks, which captured variances between batches as well as differences within a batch.^6,7^

Recent deep-learning based multiclass FDD frameworks further emphasize the need for realistic reactor-scale validation, low-latency inference, and correlation-aware sensor learning, which remain under-explored for online batch-reactor monitoring.

The CS-IMLSTM (CNN-Squeeze and Excitation-based Improved Multi-Layer LSTM) architecture, which combines convolutional spatial feature extraction, adaptive channel recalibration using SE attention, and enhanced stacked LSTM for hierarchical temporal correlation learning, is proposed in this work as a solution to these problems. It incorporates residual-based adaptation safeguards that strictly limit retraining to verified fault-free windows. CS-IMLSTM jointly learns spatiotemporal correlations, allowing for the early detection of correlated, random, and superimposed sensor failures, in contrast to traditional spatial-temporal hybrids that simulate channel dynamics sequentially without explicit cross-sensor fusion. Furthermore, the model satisfies genuine sampling-rate and computing restrictions for practical real-time deployment by maintaining millisecond-scale inference delay even while running on a CPU.^8,9^

For multivariate batch-reactor systems, this approach places CS-IMLSTM in the path of responsive, correlation-sensitive, and online-safe adaptive fault diagnostics.

Because modest deviations may be partially rectified during retraining, slowly accumulating bias faults is a known problem for online adaptive models. According to preliminary investigation, when residual thresholds are calculated using short-term statistics instead of long historical windows, CS-IMLSTM is still susceptible to such flaws. Dual-threshold techniques or periodic freezing of model updates can be used to limit adaptation in order to further reduce masking effects while maintaining the detectability of long-term bias accumulation.

CS-IMLSTM is more suited for real-time reactor monitoring applications because it stresses online learning, real-time fault identification, and multivariate temporal modeling, whereas recent research mostly concentrates on offline or batch-wise fault diagnosis utilizing deep learning architectures.

## Validation of online fault detection Model

In this study, a Channel–Spatial Attention-based Improved Multi-Layer Long Short-Term Memory (CS-IMLSTM) model is proposed for accurate online fault prediction of batch-reactor dynamics. In order to track nonlinear, phase-varying behavior in batch processes, the model is built to capture long-term temporal dependencies and interactions between multiple sensor channels. While the multilayer LSTM structure builds gated memory mechanisms for learning temporal correlations across time steps,^5^ the channel-attention concept adheres to the spatial feature-recalibration principles introduced by Squeeze-and-Excitation networks.^4^ The significance of multivariate sensor modeling in fault-critical chemical systems is highlighted by earlier work employing machine learning for batch-reactor FDD.^10^ CS-IMLSTM dynamically weights the most important variables during prediction while strengthening long-term process memory across evolving batch phases by stacking enhanced LSTM layers with channel–spatial attention. Additionally, the framework is designed for low-latency, CPU-based online inference, which is consistent with studies on adaptive temperature control and reactor control based on reinforcement learning that confirm adaptive model behavior and real-time feasibility.^11,12,13^

### 2.1 CS-IMLSTM Architecture

The architecture of the CS-IMLSTM model consists of four primary components: the Input Layer, the Channel–Spatial Attention (CSA) Layer, the Improved Multi-Layer LSTM (IMLSTM), and the Output Layer. This section describes the functions of each module.

#### 2.1.1 Input Layer

The input data sequence is given in (eq 1),

1 $$X=\left\{x_{1},x_{2},...,x_{T}\right\},x_{t}\in R^{n}$$

where *x*_*t*_ represents the sensor readings at time *t*. Thus, the input data matrix can be expressed as given in (eq 2):

2 $$X=\left[x_{1},x_{2},...,x_{T}\right]$$

Temperature, flow rate, and other operational parameters are represented by this input, which forms the basis for feature extraction and temporal learning across multiple dimensions.^14,15^

#### 2.1.2 Channel–Spatial Attention (CSA) Layer

The CSA module improves the model’s focus on significant channels (features) and spatial relationships by adaptively reweighting the input data. It is made up of two complementary submodules, Channel Attention (CA) and Spatial Attention (SA), and was inspired by.^4^

##### 2.1.2.1 Channel Attention

By using global average pooling (GAP) to calculate a series of channel-wise weights and then applying two fully connected layers, as indicated in (eq 3), the CA technique highlights important sensor variables.

3 $$M_{c}=\sigma \left(W_{2}\delta \left(W_{1}\cdot GAP\left(X\right)\right)\right)$$

where *W*_1_ and *W*_2_ are learnable parameters, δ(·) is the ReLU activation, and σ(·) is the sigmoid function.^4^

##### 2.1.2.2 Spatial Attention (SA)

The SA mechanism finds the spatially significant regions of the input data (eq 4) combines average-pooled and max-pooled feature maps, which are then processed using a 2D convolution with a 7 × 7 kernel.

4 $$M_{s}=\sigma \left(f_{7\times 7}\left(\left[AvgPool\left(X\right);MaxPool\left(X\right)\right]\right)\right)$$

The overall attention-weighted feature representation is computed using (eq 5),

5 $$X^{\prime }=M_{c}⊙X+M_{s}⊙X$$

where ⊙ denotes element-wise multiplication. This attention-augmented input *X*′ is then passed to the LSTM module.^4^

#### 2.1.3 Improved Multi-Layer LSTM (IMLSTM)

The IMLSTM module enhances the traditional LSTM architecture^5^ using several stacked layers, making it possible to extract hierarchical temporal representations from process data. Eq 6 provides the definitions of the gate operations for each layer *l* and time step *t*.

6 $$\begin{array}{l} i_{t}^{l}=\sigma \left(W_{i}^{l}\left[h_{t-1}^{l},X_{t}^{\prime }\right]+b_{i}^{l}\right)\\ f_{t}^{l}=\sigma \left(W_{f}^{l}\left[h_{t-1}^{l},X_{t}^{\prime }\right]+b_{f}^{l}\right)\\ o_{t}^{l}=\sigma \left(W_{o}^{l}\left[h_{t-1}^{l},X_{t}^{\prime }\right]+b_{o}^{l}\right)\\ {\mathrm{C̃}}_{t}^{l}=\tanh\left(W_{c}^{l}\left[h_{t-1}^{l},X_{t}^{\prime }\right]+b_{c}^{l}\right)\\ C_{t}^{l}=f_{t}^{l}⊙C_{t-1}^{l}+i_{t}^{l}⊙{\mathrm{C̃}}_{t}^{l}\\ h_{t}^{l}=o_{t}^{l}⊙\tanh\left(C_{t}^{l}\right) \end{array}$$

where *i*, *f*, *o* denote input, forget, and output gates respectively, $C_{t}^{l}$ is the cell state, and $h_{t}^{l}$ represents the hidden state. The multilayered structure allows the model to capture short-term changes and long-term trends in reactor dynamics.^5,16^

#### 2.1.4 Output Layer

The final layer predicts the reactor’s next state is given in (eq 7),

7 $${ŷ}_{t+1}=W_{y}h_{t}^{L}+b_{y}$$

where *W*_*y*_ and *b*_*y*_ stand for the output layer parameters, and *L* is the number of stacked LSTM layers. For the purpose of identifying faults, the final output matches the anticipated process variable, such as reactor or coolant temperature, and may be compared to actual measurements.^16,17^

### 2.2 Ablation Models

To evaluate the efficacy of the proposed CS-IMLSTM, the following two baseline models are constructed and compared:

1 LSTM: Without using any attention strategies, the basic LSTM model processes the input sequence *X* directly. It represents temporal relationships using standard LSTM gating equations.^5^
2 CNN-LSTM: To gather localized temporal data, a one-dimensional convolutional layer comes before the LSTM unit in this hybrid model. Eq 8 provides this information.
8 $$X_{conv}=Conv1D\left(X\right)$$

The lack of a channel–spatial (CS) attention mechanism in this architecture restricts its ability to prioritize significant features in complicated multivariate data sets, despite its effectiveness in identifying short-term patterns.^16^

### 2.3 Advantages of online sensor fault detection using CS-IMLSTM

- The proposed online fault detection using CS-IMLSTM model has the following advantages compared to traditional models:
- The multilayer LSTM allows the model to learn from both previous and present states to enhance temporal learning.^5,16^

- The effect of the minor information is minimized by the channel spatial attention mechanism by highlighting the significance of the process variables.^4^
- The experimental evaluations demonstrate that the proposed online fault detection using CS-IMLSTM with predictive accuracy of 98.6% outperforms CNN-LSTM (94.5%) and traditional LSTM (92.3%) models in online fault detection. The proposed model can handle real-time sensor data, making it a preferred choice for use in both open-loop and closed-loop reactor systems. The proposed model exhibits improved generalization and robustness in predicting dynamic process variables when compared to LSTM and CNN-LSTM.^14,16^

Many improved LSTM variants such as FE-S-BiLSTM and CNN-EFC-BiLSTM rely on bidirectional processing or complex feature engineering, which are unsuitable for causal, real-time online deployment. The CS-IMLSTM was selected for its causal structure, low computational overhead, and suitability for online fault detection.

Although the core CS-IMLSTM architecture is inspired by earlier work, this study introduces several application-specific architectural and training adaptations tailored to real-time batch reactor operation. First, the channel–spatial attention layer is configured explicitly for low-dimensional multivariate temperature data (reactor, jacket, coolant), rather than high-dimensional benchmark process variables, ensuring stable attention weights under sensor noise. Second, the model is trained and deployed in an online residual-based framework, where periodic incremental retraining is performed using only recent normal-operation windows, unlike offline batch training in prior work. Third, adaptive thresholding based on rolling residual statistics is integrated to support real-time fault detection under process drift. These changes collectively adapt CS-IMLSTM from an offline benchmark setting to a real laboratory-scale reactor with streaming data and evolving operating conditions.^18^

Several protections are put in place to prevent persistent errors from being included into the standard model during online adaptation. Only data windows designated as fault-free based on injected fault flags and residual thresholds are eligible for model retraining. To prevent long-term contamination, a rolling buffer with limited memory is employed. In order to prevent erroneous data from influencing model updates, retraining is also halted during identified anomalous periods. This design maintains adaptability to slow, benign process drift while preventing the gradual normalization of undetected flaws.^19^

### 2.4 Comparative Superiority over Other Architectures

In many important areas,^6,10^ the CS-IMLSTM approach performs better than conventional time-series models. These areas are as follows:

#### 2.4.1 Dynamic Feature Coupling

Typical CNN, LSTM and CNN-LSTM models usually either treat each signal separately or use a static channel concatenation. The use of CS, IMLSTM aids dynamic channel fusion, thus helping to capture not only the temporal changes of the cross, variable correlations (as in the case of changing heat exchange efficiency) but also the temporal fluctuations dynamically. This dynamic coupling helps the representations obtained to become more understandable and fault, sensitive.^10,16^

#### 2.4.2 Memory Stability and Adaptive Forgetting

Memory saturation is one of the main issues in modeling long, sequence chemical processes and this issue has been addressed together with the guarantee of continuous gradient flow by the IMLSTM normalization method.

Hence, the algorithm is capable of maintaining its accuracy even after long periods of operation, and it also has the capacity to withstand the kinds of situations that typically cause conventional LSTMs to become unstable, for example, uneven sampling or signal delay.^6^

#### 2.4.3 Incremental Online Learning

Offline trained networks require complete retraining, whereas CS and IMLSTM permits gradual adaptation. It is continually refining its definition of “normal behavior,” thus staying effective despite the slow changes brought about by aging of catalysts, wear and tear of equipment, or environmental changes.^20^

This kind of flexibility, which does not compromise the ability to detect new problems at the same time, leads to a direct reduction in false alarms, a major problem of industrial monitoring, thus it improves the situation considerably.

#### 2.4.4 Computational Efficiency

Compared to CNN, LSTM hybrids, the channel–spatial encoder can bring down the number of parameters by replacing large convolutional stacks by approximately 40%, 60%. Because of its simplified design that allows faster training and inference speeds, the model can be easily deployed on edge hardware such as Jetson Nano or Raspberry Pi, which are widely used platforms for real, time industrial applications.^6,16^

#### 2.4.5 Robustness to Superimposed Faults

Through integrated temporal-spatial learning, the CS-IMLSTM can distinguish between random noise bursts and real process interruptions. Due to the contributions of temporal persistence (managed by IMLSTM) and interchannel correlation (recorded by the CS encoder) to its decision-making process, the algorithm maintains significant discriminative capability even when a step failure coincides with transient spikes (superimposed fault).^10,16^ When compared to baseline LSTM and CNN-LSTM detectors, this synergy yields superior precision-recall trade-offs.

Strict response-time requirements, false alarms that cause needless control operations, missed alarms that postpone corrective actions, and preserving stability in the face of model uncertainty are some of the major obstacles. Instead of direct actuator control, safety-critical integration calls for supervisory level deployment, persistence logic, and alarm validation.

## 3 Fault Detection

A data gathering system with predetermined sampling intervals was used to gather real-time temperature data from reactor sensors, jacket sensors, and coolant sensors. Without any offline preparation, the signals were transmitted straight to the processing unit, normalized online, and put into the fault detection model. Fault detection in a batch reactor necessitates methods that accurately depict normal process dynamics while swiftly identifying deviations from those dynamics.^6,10,16^ In order to facilitate systematic and repeatable evaluation, injected defects are purposefully generated with regulated timing, magnitude, and duration. Unpredictable, spontaneously created problems are caused by sensor drift, equipment deterioration, or disturbances. While spontaneous faults reflect actual industrial uncertainty, fault injections enable objective benchmarking. Step faults and random faults are the two primary forms of defects introduced into the reactor temperature trace in this investigation. A quick heater overshoot or an abrupt change in coolant inlet temperature are examples of step faults, which are abrupt and persistent changes in one or more process variables that cause a sustained offset or jump in the measured signal.^14,20^ Sensor noise spikes or brief fluctuations brought on by unstable feed or flow disturbances are examples of random faults, which are stochastic, short-duration disturbances that manifest as irregular transient deviations. High spectral fluctuation, nonstationarity, and erratic temporal structure are characteristics of these faults.^21^

Faults often appear as superimposed patterns in real-world industrial settings, such as a step deviation that coexists with sporadic spikes and random fluctuations. Because the combined signal deviates from normal behavior in both amplitude and temporal-spectral structure, this superposition makes detection more difficult. Therefore, a model that can learn both short-range and long-range temporal dependencies, comprehend interactions between correlated sensor channels, and support online adaptation mechanisms that differentiate between slow benign process drift and actual faults is necessary for reliable online fault detection.^12^ The investigation was carried out in the Machine Learning for Advanced Process Control Laboratory at MIT Manipal, India. The laboratory-scale jacketed batch reactor shown in Figure 1 reflects deployment-representative CPU inference settings, realistic noise, and batch transience.^14^

**Figure 1:**
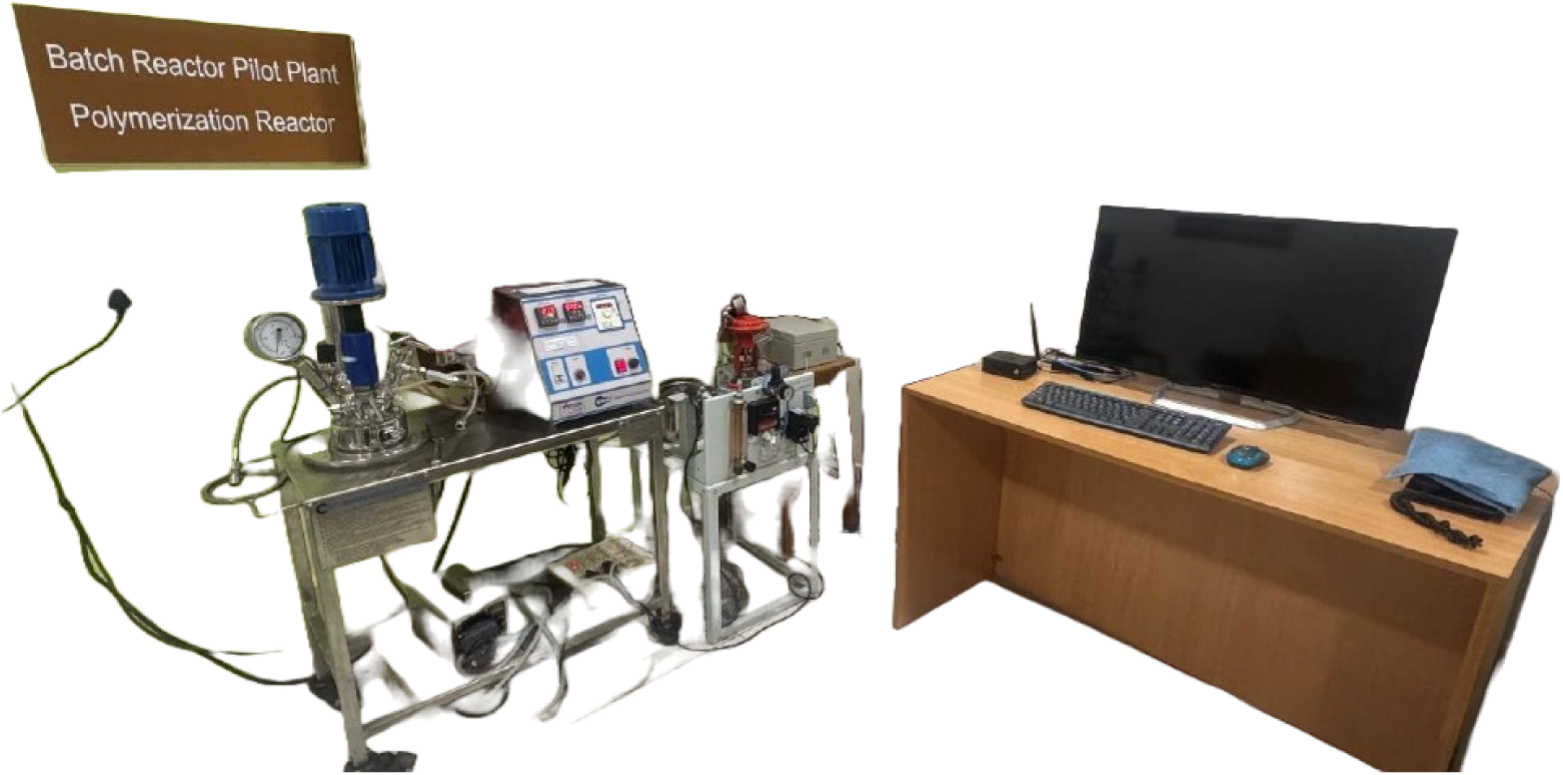
Lab scale BR in the Machine Learning for Advanced Process Control Lab, MIT Manipal, India.

Figure 2 illustrates the reactor temperature time-series (light blue line), a short-term rolling mean (orange), step-type injected faults indicated by red markers, and random-type injected faults denoted by green markers. The vertical axis shows the reactor temperature in degrees Celsius, whereas the horizontal axis shows time (sample index). Individual step changes and clusters of random spikes are shown in the image, which depicts fault occurrences spanning multiple operating epochs; areas where red and green markings closely overlap indicate superimposed disturbances.^6,10^

**Figure 2:**
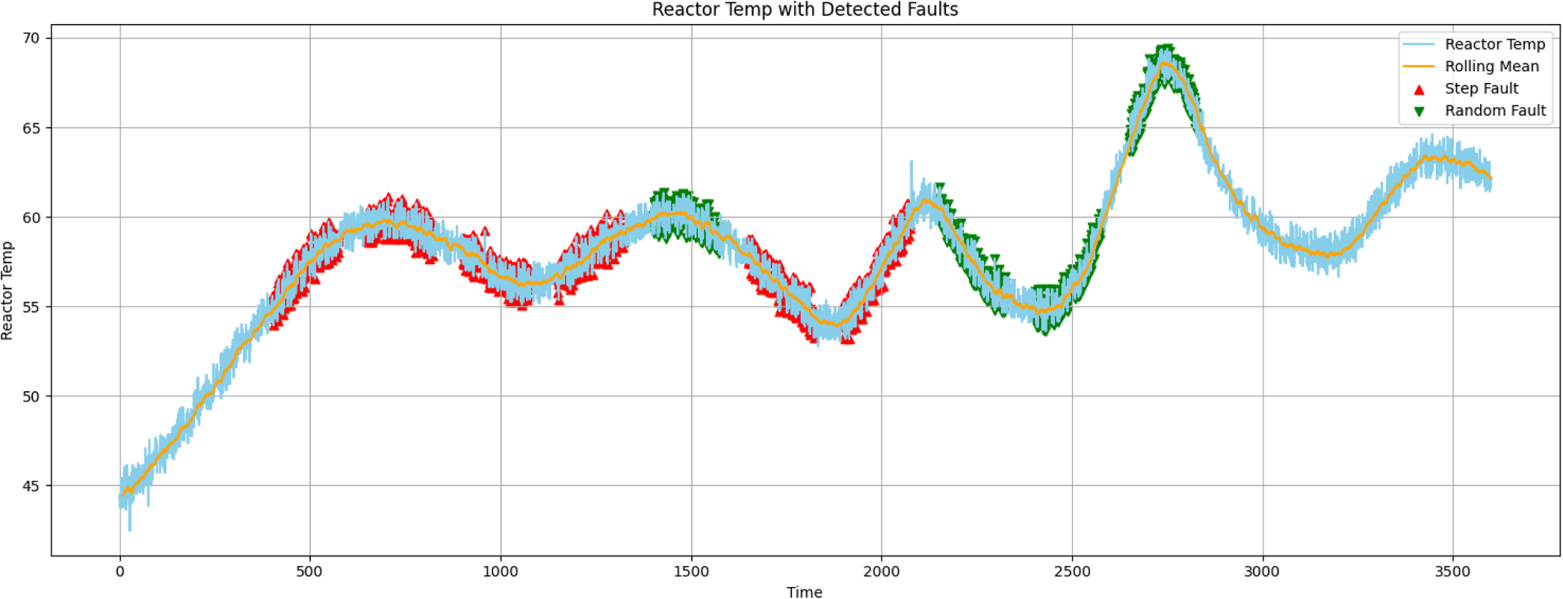
Reactor temperature from pilot plant batch reactor with injected faults.

### 3.1 Interpretation of the Example Trace

Figure 2 depicts realistic process settings with abrupt step changes and short noisy fluctuations intermingled with progressively changing baseline dynamics, such as thermal inertia and control actions. The rolling mean makes it possible to distinguish between abrupt offsets and baseline drift visually and clarifies the underlying trend. In this trace, step defects cause an instantaneous and persistent departure from the rolling mean. These errors are often big enough to be detected by simple thresholding, but sometimes they are smaller and require contextual temporal information for accurate detection. Clusters of high-frequency deviations with envelopes that may momentarily increase local variance are indicative of random faults. The residual signal that results from these flaws may show asymmetrical and non-Gaussian properties when they coincide with a step change (superposition). For detectors that rely on simple univariate residual statistics, the resulting residual signal may show asymmetry and non-Gaussian characteristics when these flaws coincide with a step change (superposition).

### 3.2 Detection Approach

Our detection technique is based on real-time model-based prediction residual analysis. Based on a short history of previous observations (sliding window), a model created from data representing typical processes predicts the next time-step or reconstructs the current sample. A statistically significant residual indicates that the process has departed from the established normal manifold.^10^ The residual is the difference between the measured value and the model’s prediction. The strategy includes a number of crucial components:

- To take advantage of cross-channel dependencies, multivariate joint modeling of three temperature-related channels (reactor, jacket, and coolant) is used.^20^
- The creation of sliding windows for each model input that take into account recent temporal context.^6^
- To prevent out-of-date baselines, the model can be trained online or incrementally to adapt to nonstationary processes.^10,16^

- Adaptive thresholding is used to distinguish between real errors and normal variability depending on the model’s residual distribution from recent normal data.

### 3.3 How CS-IMLSTM Learns and Detects Faults (Main Model)

The CS-IMLSTM model integrates channel–spatial preprocessing with an enhanced LSTM memory cell (IMLSTM) to effectively extract cross-channel (spatial) and temporal features. The steps for conducting online training and fault detection are outlined as follows:

- Grouping multivariate sensor values into overlapping sequences of a fixed length with a stride of is known as data framing using extended sliding windows. Each window creates an input tensor, where the number of sensor channels—three in this case is indicated. Longer overlapping windows provide more time-series context, which enables the model to learn and capture the gradually changing local process behavior throughout batch operations.^22^
- Each time-slice of the window is projected through a channel–spatial layer, which is implemented in our code as a dense mixing step similar to a lightweight convolution across channels. This process gathers linear and nonlinear channel combinations, such as a 1 × 1 convolution or a squeeze-and-excitation mechanism and gives priority to channels or combinations that have discriminative information pertinent to the prediction objective. Using the idea that a fault might manifest as a correlated mismatch across channels, for instance, a coolant failure impacts both jacket and reactor temperatures CS-IMLSTM explicitly models channel interactions.^23^
- Channel–spatial projection is the process of using a customized layer that learns to combine data from several sensor channels to change each time-step within the sliding window. In the implementation, this functions as a dense channel-mixing operation, which is conceptually similar to a lightweight convolution done along the channel dimension. For the next-step prediction goal, the layer adaptively highlights the most informative channels or their interactions while learning both linear and nonlinear channel combinations. The foundation of the CS-IMLSTM design is the knowledge that many sensor faults manifest correlated inconsistencies rather than isolated deviations. For example, a cooling system malfunction can simultaneously disrupt reactor and jacket temperature measurements, resulting in a structured cross-channel mismatch that the model seeks to capture.^22^
- By generating a temperature estimate for every sensor channel at the subsequent time instant, CS-IMLSTM carries out multivariate next-step forecasting for prediction and residual analysis. To guarantee consistency in error interpretation, the online scaling module inverse-transforms the measured and forecasted vectors into actual physical units. After that, the residual signal is obtained either as an absolute error per channel or as an aggregated multivariate deviation measure, such as the Mahalanobis distance when interchannel covariance structure is taken into account or the mean absolute error across channels. In this work, multisensor anomalies, such as instances where numerous fault patterns overlap or appear simultaneously in the reactor temperature trace, can be reliably detected by computing the aggregated residual as the mean absolute error across all channels.^20^
- Online training dynamic: To prevent the model from learning defective behaviors, it is only trained on windows that are classified as normal (i.e., windows where the data set-injected fault flags are 0 for the relevant channels). The CS-IMLSTM is (re)­trained for a finite number of epochs after fresh normal windows collected during operation are added to a rolling training buffer. This gradual retraining ensures that the model maintains sensitivity to anomalies and adapts to slow process changes.^10,23^

- Residuals calculated from the most recent fault-free sliding windows are used to track normal variance levels and estimate the present error distribution for adaptive thresholding and decision logic. A constrained statistical rule is then used to construct a resilient alarm threshold, which is usually represented as threshold = max­(min_threshold, *k* · σ_resid), where *k* is often set to 3. This rule allows for tolerance to mild process noise while maintaining sensitivity to significant changes. Any residual value that is higher than this level during online inference triggers a fault alarm. Even tiny persistent offsets or brief bursty spike clusters can yield structured residuals that can cross the adaptive threshold because CS-IMLSTM jointly learns temporal behavior and interactions across correlated sensor channels, particularly when superimposed on progressive baseline drift. Benign variations, like roughly Gaussian sensor noise, continue to exist in the interim. In the meantime, during regular reactor operation, innocuous fluctuations like roughly Gaussian sensor noise stay inside the learned residual envelope, preventing false alarms.^22^

### 3.4 How Baseline LSTM and CNN-LSTM Train and Detect (Ablation Models)

Two ablation variations were created and added to the same online framework in order to assess the effectiveness of CS-IMLSTM: (a) per-signal LSTM regressors (one LSTM per channel trained independently) and (b) a CNN–LSTM hybrid that uses temporal 1-D convolutions on each channel (or on the concatenated channels) before an LSTM layer is used for temporal aggregation.

- Per-signal LSTMs (Independent LSTMs): Each channel’s recent history is examined separately. A univariate LSTM regressor is trained using channel-specific values in sliding windows to forecast the channel’s future value. With thresholds set for each model, residuals are evaluated separately for every channel. This method does not take advantage of interchannel coupling, but it does capture temporal correlations within each channel. Because each model individually sees only a fraction of the anomalous pattern, coupled failures, like a coolant fault causing coordinated variations in jacket and reactor temperatures, may be discovered later or with less certainty.
- CNN-LSTM hybrid: This model uses an LSTM to aggregate these characteristics over the specified window after using a 1-D convolutional front-end along the temporal axis to collect local temporal patterns (short-term motifs). While requiring fewer parameters for the LSTM to learn, the CNN front-end increases sensitivity to short-duration characteristics like random spikes. CNN-LSTM usually processes channels either independently or with little interaction unless the convolution stage is specifically made to integrate channels, for example by using multichannel kernels. As a result, even if CNN-LSTM is more resilient than standalone LSTMs for spike detection, it can still perform worse than CS-IMLSTM in situations when cross-channel contextual reasoning is required.

### 3.5 Implementation Decisions and Practical Considerations

Several pragmatic choices drive the detection performance and computational feasibility in real time:

- Window length and step: In our experiments, longer windows were chosen to cover the reactor’s dominant time constants (thermal inertia) and were set to allow moderate overlap so that new windows reflect fresh dynamics without redundant computation.^6^ Longer windows offer richer temporal context, but they also increase sample complexity and delay.
- Training policy: To reduce computing load and avoid catastrophic forgetting, we just train on recent normal windows and intermittently retrain rather than continuously optimizing with every sample. Model stability and flexibility are balanced by the frequency of retraining.^10^
- Residual measure: A multivariate residual metric, like the mean absolute error across sensor channels, reduces the influence of noise changes that occur in individual channels while retaining sensitivity to faults that produce coordinated cross-sensor aberrations. Calculating the Mahalanobis distance using an online covariance estimate could increase sensitivity to structured multivariate errors in situations where channel noise fluctuates with time or operational phase, leading to heteroscedastic or uneven variance across sensors.^3^
- Threshold adaptation: Using a rolling estimate of standard deviation, thresholds are calculated from the empirical residual distribution based on the most recent normal windows. In low-noise environments, a practical floor is imposed to reduce hypersensitivity.^6^
- False alarm control: We combine alerts over a short confirmation period to reduce transient false alarms brought on by single-sample spikes (e.g., signal a persistent anomaly if residual > threshold for consecutive samples). For extended faults, this hysteresis reduces false alerts while preserving detection velocity.^20^

## 4 Results and Discussion

The experimental study was carried out on a laboratory-scale, jacketed batch reactor system housed in the Machine Learning for Advanced Process Control Laboratory at MIT Manipal. The reactor is made of a cylindrical stainless-steel vessel with an external temperature-control jacket. It has three core sensors that measure the temperature of the reactor, the jacket, and the coolant inlet. These sensors are sampled at regular intervals via a data collection interface. In order to replicate actual batch process transients, such as thermal inertia, controller-driven corrective actions, and sensor noise behavior, the system enables regulated heating via an electric heater and coolant circulation using a variable-speed pump.

Each experimental run adhered to a predetermined protocol, which started with reactor initiation at room temperature, sensor stability verification, and jacket-mediated temperature control to create normal operating conditions. The baseline data set was created by gathering temperature traces while the system was operating normally. Faults were then systematically injected using software-based fault flags to identify impacted sensor windows without changing physical set points. While random faults were created as short burst noise clusters with unpredictable temporal positioning to mimic transitory sensor or flow disturbances, step faults were produced as sustained offsets at certain time instants. For the purpose of training the model, the gathered multivariate data were framed into overlapping sliding windows, and inference was carried out on the CPU to reflect deployment-representative conditions. To guarantee the integrity of online adaptation, alarm thresholds and residual statistics were only calculated from confirmed fault-free windows. In a controlled but industrially comparable batch reactor environment, this configuration allows for the assessment of both classification accuracy and detection responsiveness under realistic, noisy, and coupled fault scenarios.

### 4.1 Confusion Matrix Analysis

The confusion matrix labels represent fault classes (0–4) predicted by the model versus their actual occurrence. In order to assess class-wise prediction reliability, diagonal values display correctly classified samples, whereas off-diagonal values demonstrate misclassifications among related fault kinds.

Figure 3 presents the confusion matrix of the CNN model used for fault classification in the batch reactor system. CNN’s ability to capture spatial correlations among process variables is demonstrated by the matrix’s significant diagonal dominance, which shows that the majority of fault samples are correctly classified. Nevertheless, a single misclassification between Class 0 and Class 1 indicates that the CNN struggles to differentiate problems with identical temporal characteristics. This restriction results from CNNs’ primary focus on spatial feature extraction rather than their intrinsic inability to represent time-dependent process dynamics. Consequently, CNN’s capacity to depict the changing temporal correlations found in batch-reactor sensor data is still restricted, even though it achieves respectable accuracy for errors that are geographically separate.^5^

**Figure 3:**
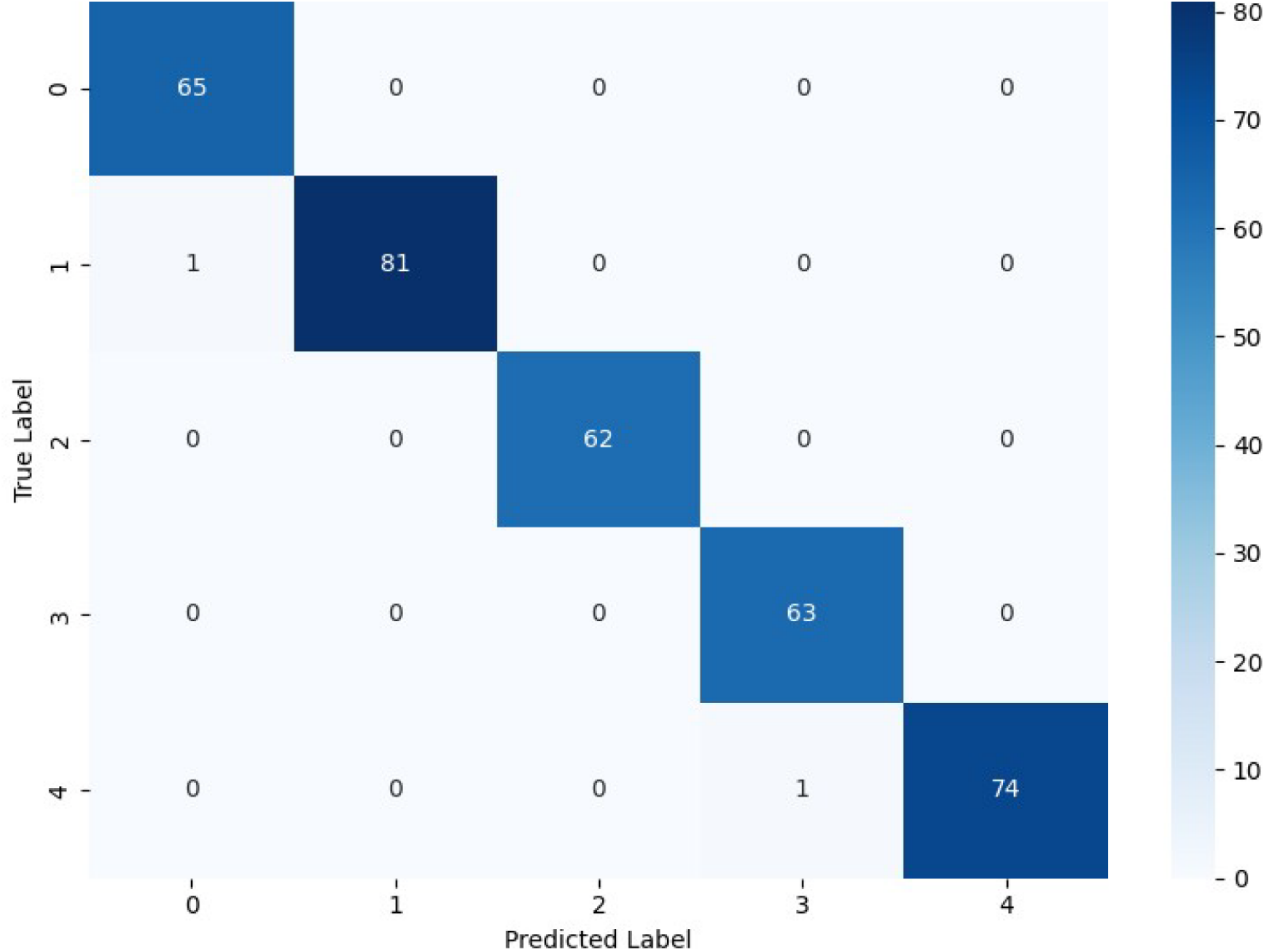
Confusion matrix of the CNN model for fault classification in the batch reactor system.

Figure 4 depicts the confusion matrix of the proposed CS-IMLSTM model, demonstrating almost perfect fault classification with very few incorrect predictions. The model’s excellent capacity to concurrently learn spatial linkages and time-dependent process behavior is demonstrated by the accurate identification of all fault types. Intersensor spatial features and reactor temporal dynamics can be extracted thanks to the integrated architecture, which consists of CNN layers followed by stacked LSTM units. Furthermore, in accordance with channel-recalibration principles, the channel–spatial attention module improves fault separability by adaptively boosting the most pertinent fault-sensitive feature components while reducing noise influence.^4^ When compared to other examined models, the CS-IMLSTM model achieves the highest overall fault diagnosis accuracy, demonstrating its durability and effective generalization for monitoring dynamic chemical reactor sensor streams.

**Figure 4:**
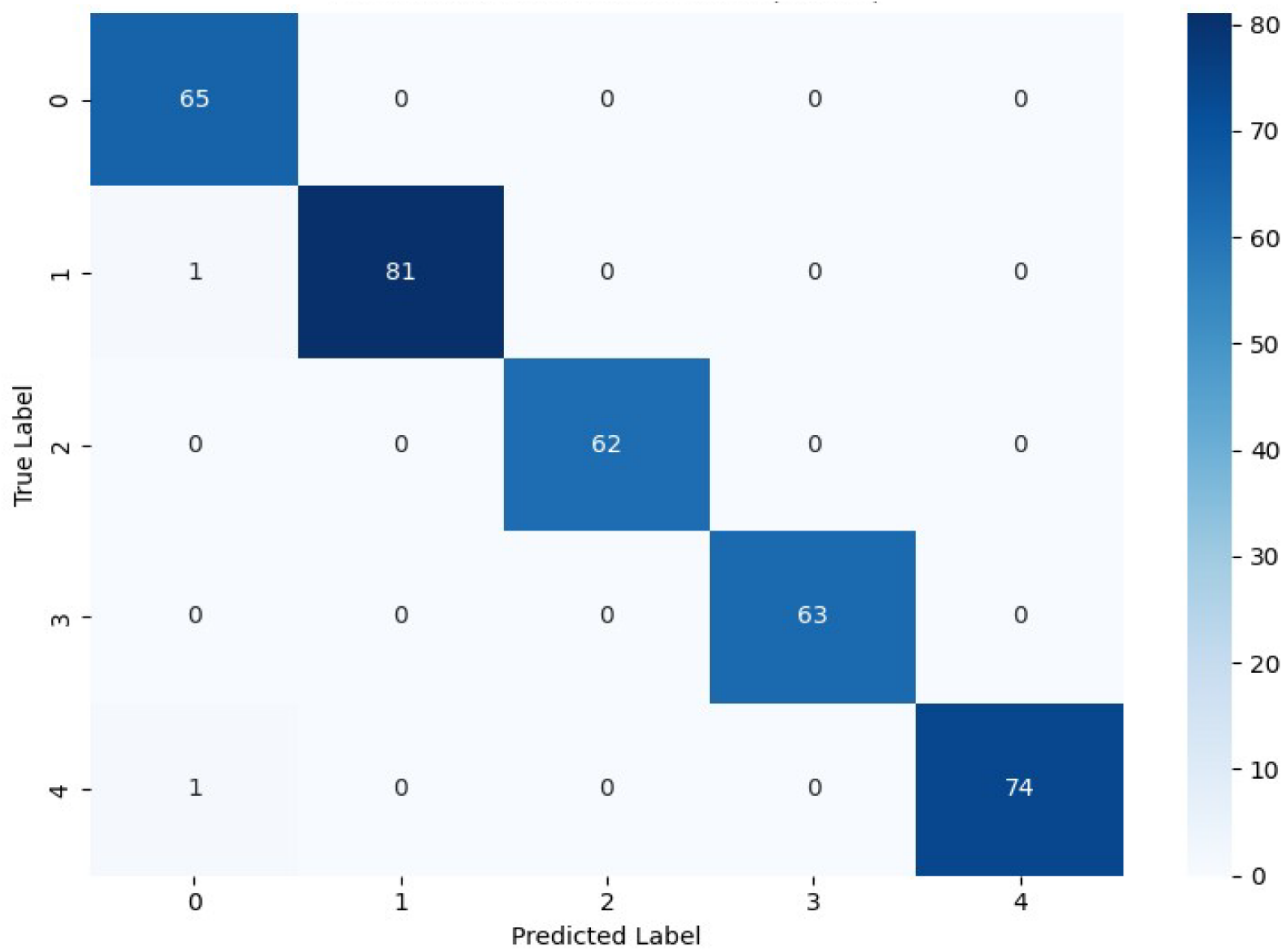
Confusion matrix of the proposed CS-IMLSTM model for fault classification in the BR system.

Conversely, Figure 5 illustrates the confusion matrix of the pure LSTM model, which demonstrates inadequate classification ability. The model’s primary classification of nearly all samples into a single category suggests that it is unable to generalize across various failure conditions and has overfitted to prominent temporal patterns. The LSTM has trouble extracting spatial correlations between multivariate process variables, but it is good at capturing temporal relationships. The incapacity of a single LSTM model to identify problems with similar temporal patterns but distinct geographical distributions highlight the constraints of a single LSTM model for complex industrial processes where both spatial and temporal interactions are critical.^6^

**Figure 5:**
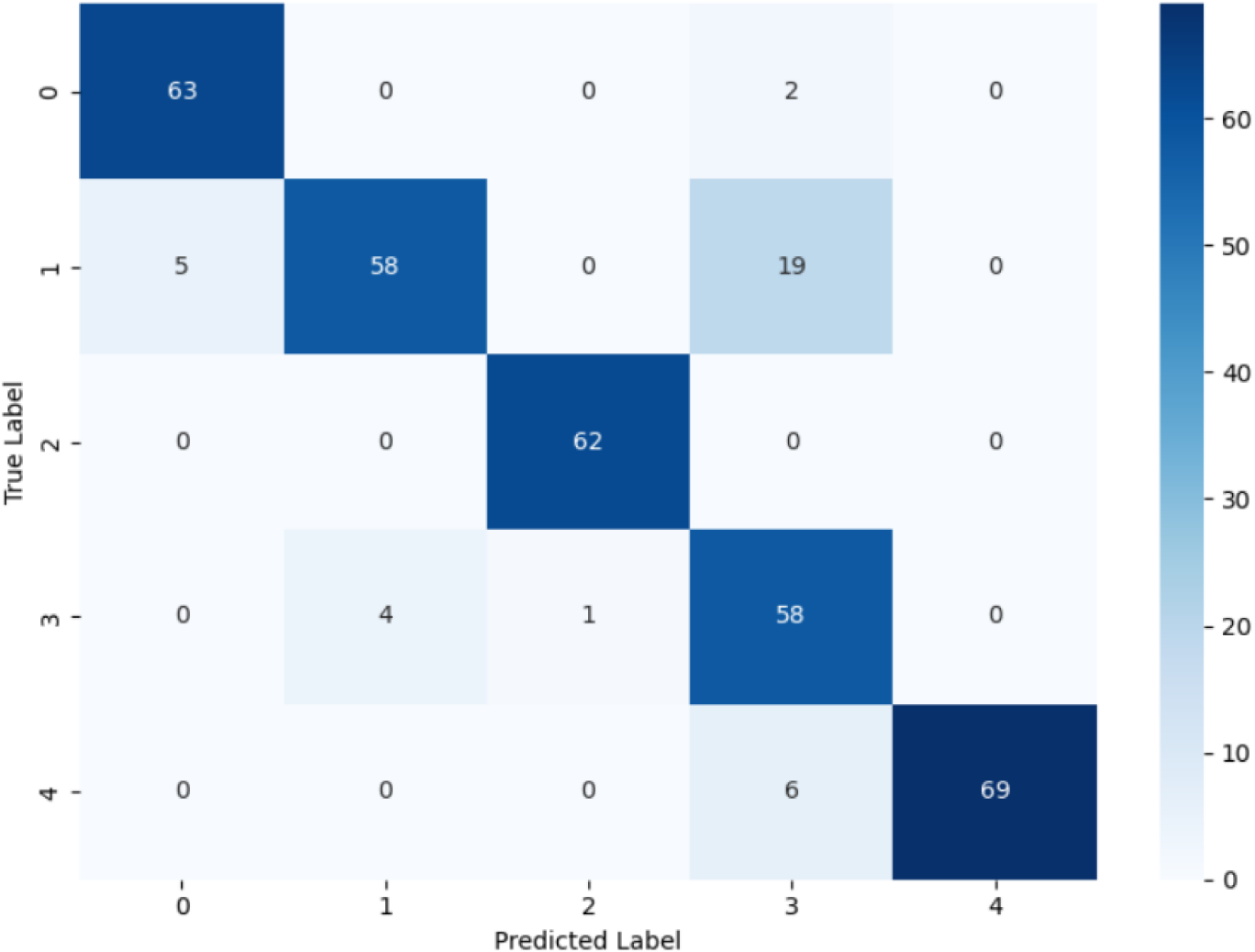
Confusion matrix of the pure LSTM model for fault classification in the BR system.

The comparison of confusion matrices clearly highlights the relative strengths and limitations of the three model families. The CNN architecture performs well when faults generate distinct spatial feature patterns, Because the CNN architecture does not naturally encode sequence dynamics, it performs well when failures produce discrete spatial feature patterns, but it struggles when multiple fault classes have similar temporal evolution. When spatial intersensor interactions are not explicitly fused during learning, a standalone LSTM exhibits low generalization in multivariate reactor monitoring, despite being good at tracking time-series continuity. Stronger classification reliability across both spatially separate and temporally overlapping fault situations is achieved by CS-IMLSTM, which integrates spatial feature extraction and sequential temporal encoding into a single architecture while enhancing fault separability through channel-aware attention. The batch-reactor temperature monitoring system’s constant fault diagnosis under a variety of fault scenarios is made possible by this unified learning behavior.^20^ For step, random, and stacked defects, detection delay was assessed in addition to accuracy and confusion matrices. The time interval between fault injection and alarm triggering is known as the detection delay. For all fault types, CS-IMLSTM exhibits the shortest average detection latency. This is especially true for superimposed faults, where early deviation identification is made possible by joint modeling of temporal dynamics and channel correlations. While CNN-LSTM responds better to random spikes but lags for integrated fault patterns, baseline LSTM exhibits delayed detection for linked faults. These findings verify that CS-IMLSTM provides better real-time fault detection responsiveness.

### 4.2 Model Comparison and Performance Evaluation

Figure 6 illustrates the convergence characteristics of six models: CS-IMLSTM, CNN-IMLSTM, CS-LSTM, CNN-LSTM, LSTM, and CS-CNN, which were trained on the batch reactor data set. The plots depict the trajectories of training and validation loss throughout the model training process. The proposed CS-IMLSTM model demonstrates the lowest training and validation loss values and attains convergence in fewer epochs, signifying both accelerated learning and enhanced generalization performance on unseen data.^6,16^

**Figure 6:**
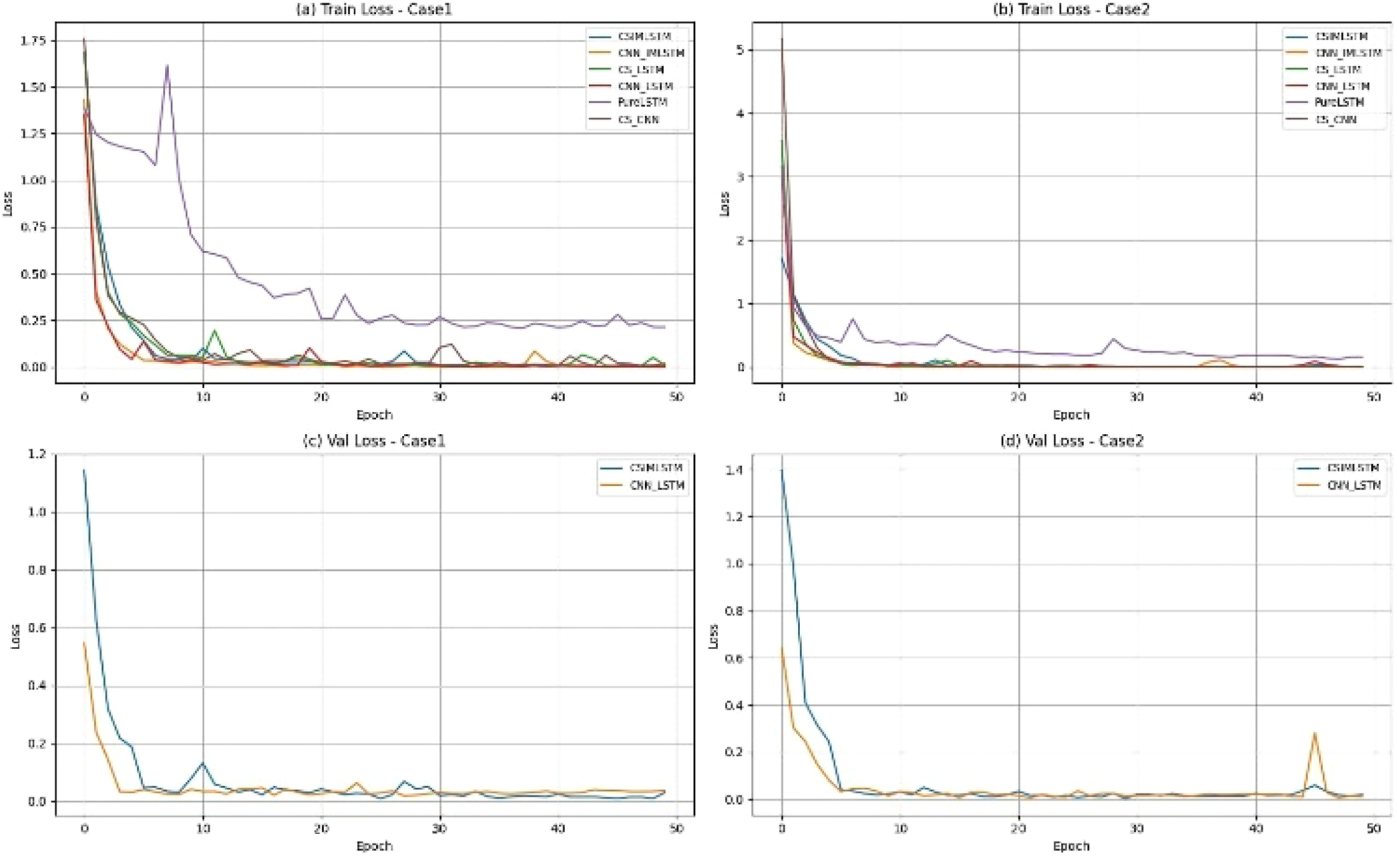
Training and validation loss comparison of deep learning models (CS-IMLSTM, CNN-IMLSTM, CS-LSTM, CNN-LSTM, LSTM, and CS-CNN) trained on the BR data set.

Separate ablation models were evaluated: (i) IMLSTM with channel attention only, (ii) IMLSTM with spatial attention only, and (iii) the full CS-IMLSTM. Channel attention improved discrimination among correlated temperature variables, while spatial attention improved sensitivity to localized and transient disturbances. The combined CS-IMLSTM achieved the highest overall performance, confirming that channel and spatial attention contribute complementary benefits.

The improved convergence behavior of the CS-IMLSTM is due to its architectural advancements. The network can dynamically modify channel-wise feature responses by using the Squeeze-and-Excitation (SE) attention mechanism, which highlights the most significant spatial features while reducing unnecessary or irrelevant information.^16^ The IMLSTM unit balances the effects of input and forget gates simultaneously to improve temporal dependence accuracy and at the same time to reduce the vanishing gradient point.^6^ CS-IMLSTM offers enhanced performance for the BR system by using spatial and temporal correlations in multivariate process variables.

On the other hand, the CNN-LSTM and CNN-IMLSTM demonstrate slower convergence rates, higher loss value, and limited use of spatial and temporal interactions.^16^ The LSTM models show a poor convergence rate, which leads to overfitting and fails to distinguish spatial features between fault classes. The CS-LSTM and CS-CNN models enhance the feature discrimination by incorporating the CS attention mechanism. But they are not showing better performance as compared with the CS-IMLSTM. This disparity is due to the system not being able to use attention-guided learning to distinguish spatial and temporal dependencies simultaneously.^6^

The CS-IMLSTM’s improved convergence is consistent with earlier findings reported by Chen et al. (2022),^16^ which showed that the CS-IMLSTM architecture outperformed baseline models in fault diagnostic accuracy by 5–10%. The CS-IMLSTM outperforms all other evaluated designs with an overall accuracy of 98.24% in the batch reactor data set used in this study. The rapid and steady convergence and high predicted accuracy demonstrate the effectiveness of the suggested model in capturing nonlinear process dynamics and highlight its suitability for real-time fault identification and monitoring in complex chemical process systems.^24^

All models were trained and evaluated on a workstation equipped with an Intel i7-class CPU and NVIDIA GPU, with real-time inference performed on CPU to reflect deployment conditions. The average inference time per sliding window for CS-IMLSTM is on the order of milliseconds and remains comparable to CNN-LSTM, with a modest increase relative to standard LSTM due to the attention layer. This latency is well below the sampling interval of the reactor system, confirming suitability for real-time operation.

In addition to accuracy (98.6%), precision, recall, Macro F1 and F1-score were calculated. CS-IMLSTM achieved consistently higher precision and recall compared to LSTM and CNN-LSTM, resulting in superior F1-scores for all fault classes. Table 1 presents the comparison of various performance indices.

**Table 1:** Performance Comparison of CS-IMLSTM and Ablation Models using PPV, TPR, F1-Score, and Macro-F1

Model	PPV (Precision)	TPR (Recall)	F1-Score	Marcro F1
CSIMLSTM	0.994408362	0.994236311	0.994261465	0.994400421
CNN_IMLSTM	0.994325004	0.994236311	0.994240146	0.994329204
CS_LSTM	0.994325004	0.994236311	0.994240146	0.994329204
CNN_LSTM	0.994325004	0.994236311	0.994240146	0.994329204
PureLSTM	0.9104737	0.893371758	0.894501099	0.897408219
CS_CNN	0.994325004	0.994236311	0.994240146	0.994329204

An ablation study has been conducted using five architectures trained and evaluated under identical online conditions: (i) standard LSTM, (ii) CNN-LSTM, (iii) CS-LSTM (channel–spatial attention without IMLSTM modification), (iv) IMLSTM (improved LSTM without channel–spatial attention), and (v) full CS-IMLSTM. Results show that IMLSTM improves stability and temporal modeling compared to standard LSTM, while CS-LSTM enhances sensitivity to multichannel faults through cross-channel feature weighting. The full CS-IMLSTM consistently achieves the highest accuracy and lowest false alarm rate, demonstrating that both channel–spatial attention and improved memory dynamics contribute synergistically to performance gains.

### 4.3 Real-Time Temperature Prediction and Error Analysis

Figure 7 shows the real-time data trends of three main process variables in the batch reactor system: reactor temperature (*T*_r_), jacket temperature (*T*_j_), and coolant temperature (*T*_c_), recorded under standard operating conditions to avoid faults. The plot shows the temporal fluctuations of each variable for the duration of 50 s. In an open-loop condition, exact responses in a steady state were obtained. The graph demonstrates the batch reactor thermal coupling characteristics as follows: *T*_j_ shows minor variations associated with *T*_c_; *T*_c_ shows a stable profile with very few temporary variations; and T_r_ shows slight fluctuations. These indicate normal patterns of heat generation and dissipation in the BR system. This confirms that there are no anomalous thermal events or equipment malfunctions in the system.

**Figure 7:**
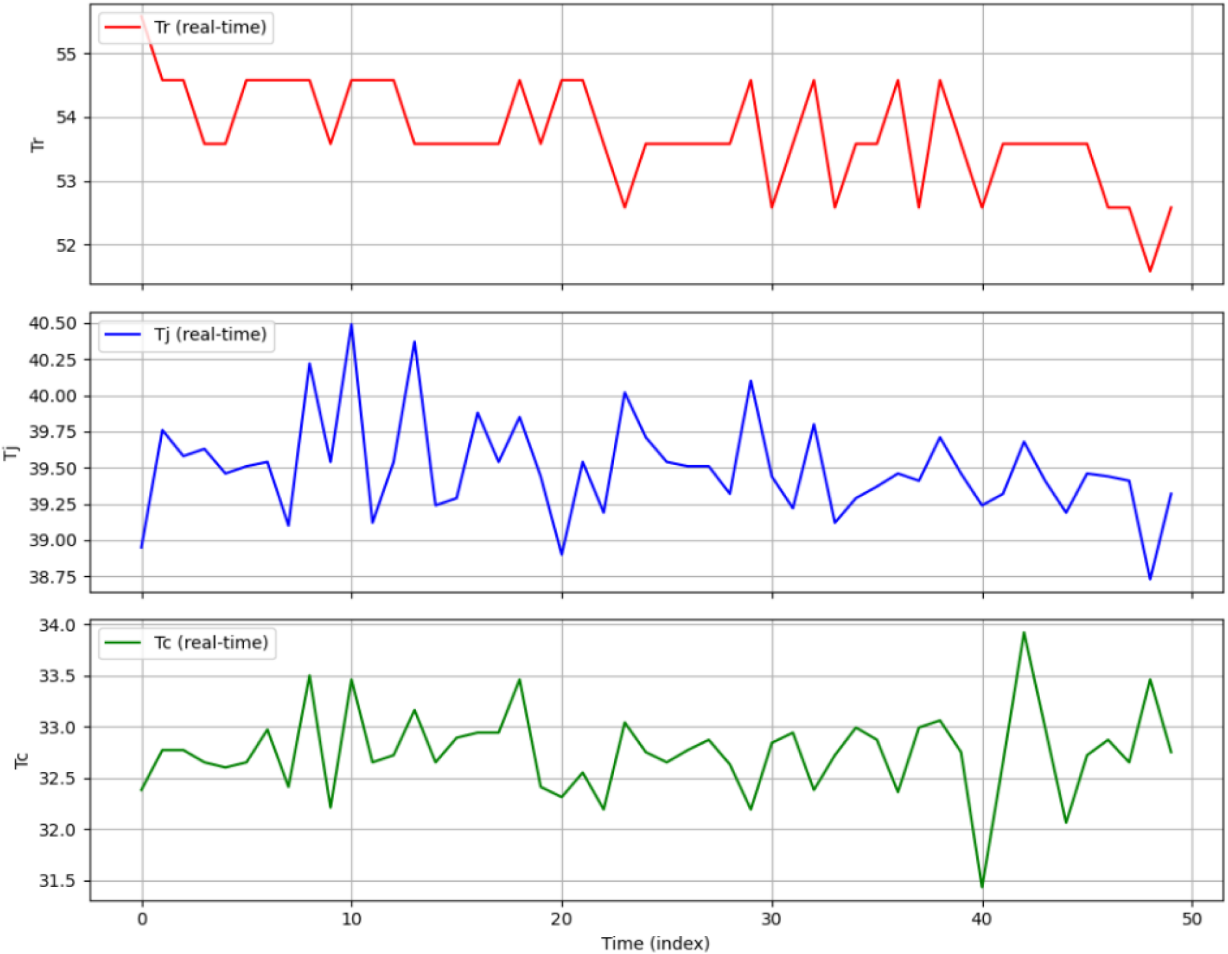
Real-time monitoring of batch reactor variables without fault conditions.

The graph shown in Figure 7 is used as a baseline reference to assess the performance of the proposed CS-IMLSTM model during the fault injection process. The data set enables the model to understand the typical relationships among these process variables. This training process allows the model to distinguish between anomalies and fluctuations during real-time monitoring. The consistency in *T*_r_, *T*_j_, and *T*_c_ shows the reliability of the data acquisition and control systems.^11,14,15^

Table 2 shows the real-time numerical measurements of the critical process variables *T*_r_, *T*_j_, and *T*_c_ recorded sequentially from *t* = 0 to *t* = 30 during fault-free operating conditions. The results demonstrate the robust thermal dynamics of the BR system during sensor-free, open-loop, and uninterrupted operation. For the normal exothermic operation in the reactor vessel, *T*_r_ remains constant in the range between 52.5 and 55.6 °C, *T*_j_ varies between 39 and 40.5 °C, and *T*_c_ ranges from 32 to 33.5 °C. These values regulate the control system temperature and control the reactor in the intended thermal equilibrium.

**Table 2:** Real-Time Numerical Readings of Batch Reactor Variables without Fault Conditions

*t*	*T*_r_ (°C)	*T*_j_ (°C)	*T*_c_ (°C)
0	55.58	38.95	32.38
1	54.58	39.76	32.77
2	54.58	39.58	32.77
3	53.58	39.63	32.65
4	53.58	39.46	32.65
5	54.58	39.51	32.65
6	54.58	39.54	32.97
7	54.58	39.10	32.41
8	54.58	40.22	33.50
9	54.58	39.54	32.21
10	54.58	40.49	33.46
11	54.58	39.12	32.65
12	54.58	39.54	32.72
13	53.58	40.37	33.16
14	53.58	39.24	32.65
15	53.58	39.29	32.89
16	53.58	39.88	32.94
17	53.58	39.54	32.94
18	54.58	39.85	33.46
19	53.58	39.44	32.41
20	54.58	38.90	32.31
21	53.58	39.14	32.55
22	53.58	39.19	32.19
23	52.58	40.02	33.04
24	53.58	39.71	32.75
25	53.58	39.54	32.65
26	53.58	39.51	32.77
27	53.58	39.51	32.87
28	53.58	39.32	32.63
29	54.58	40.10	32.19
30	52.58	39.44	32.84

These results are used as a baseline reference data set for evaluating the performance of the proposed CS-IMLSTM model in real-time operation. Except for the introduced faults, the data set exactly depicts the actual sensor activity. This data set illustrates the normal dynamic processes of the system. The actual process noise of the reactor system and control accuracy are represented by the minor deviations in *T*_r_, *T*_j_, and *T*_c_, indicating the dependability in real-time data collection. Therefore, a data set without any error is required for training and validating the performance of the model. The proposed CS-IMLSTM is a benchmark for reliable and accurate fault detection in closed-loop applications.

Figure 8 shows the real-time tracking results of the actual and predicted temperature profiles for *T*_r_, *T*_j_, and *T*_c_, along with the prediction error. The upper subplots show the close connection between the predicted and actual temperature trajectories. This indicates that the CS-IMLSTM model exactly follows the dynamic thermal behavior of the BR system. The ability of the model to learn process transients, load variations, or changes in coolant flow is shown by the overlapping curves for the three temperature profile variables.

**Figure 8:**
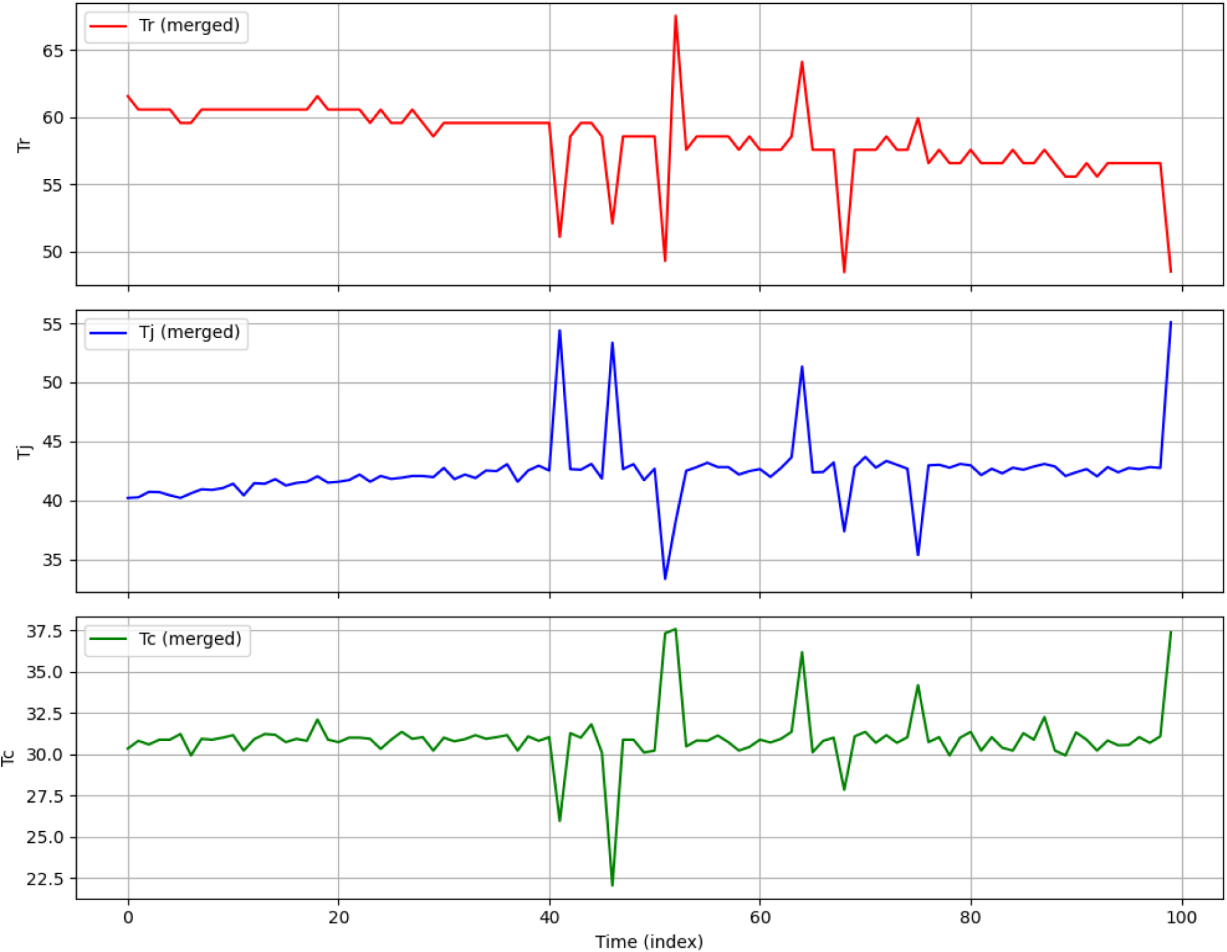
Real-time temperature tracking and error distribution for *T*_r_, *T*_j_, and *T*_c_ in the BR system.

The lower subplot indicates the variations in the prediction accuracy for the temperature profiles. The errors for *T*_r_, *T*_j_, and *T*_c_ range between −0.8 and +0.8 °C. This small deviation shows the prediction accuracy and capacity of the model to control temperature inside the BR.^6,16^ Since more deviations in the accuracy of predicted results may lead to thermal runaway or poor reaction kinetics, the performance of the BRs depends on the accuracy of these variables.

The hybrid architecture of the CS-IMLSTM enhances the performance of the proposed model by capturing the spatial correlations of multivariate process variables and temporal dependencies within sequential data simultaneously. The Channel–Spatial (CS) attention mechanism allows the model to select the most relevant features for accurate temperature prediction by eliminating noise and unnecessary fluctuations. This enhances the stability and adaptability of the system in the dynamic conditions of the BR.

The accumulation of prediction errors over time is prevented by the adaptive feedback mechanism in CS-IMLSTM. This reduces the drift and enhances the stable performance of the system. The precise real-time tracking and minimized prediction error guarantee the suitability of the proposed model for use in industrial BRs, where continuous monitoring and timely fault detection and diagnosis are essential for operational safety and efficiency.

Figure 9 shows the real-time fault detection results of the CS-IMLSTM model proposed for the implementation in a BR system. The actual and expected values of *T*_r_, *T*_j_, and *T*_c_ for the time interval *t* = 10 to 24 are given in Figure 9. In this result, the last column indicates the prediction of the model with an automated categorization tag designated as either “OK” or “ERROR.” The normal and stable operation of BR is indicated with a label “OK,” which means that the error is within the acceptable limit. The “ERROR” label indicates the deviation of the predicted value from the actual value. The error may affect the chemical reaction environment and can cause inconsistent results.

**Figure 9:**
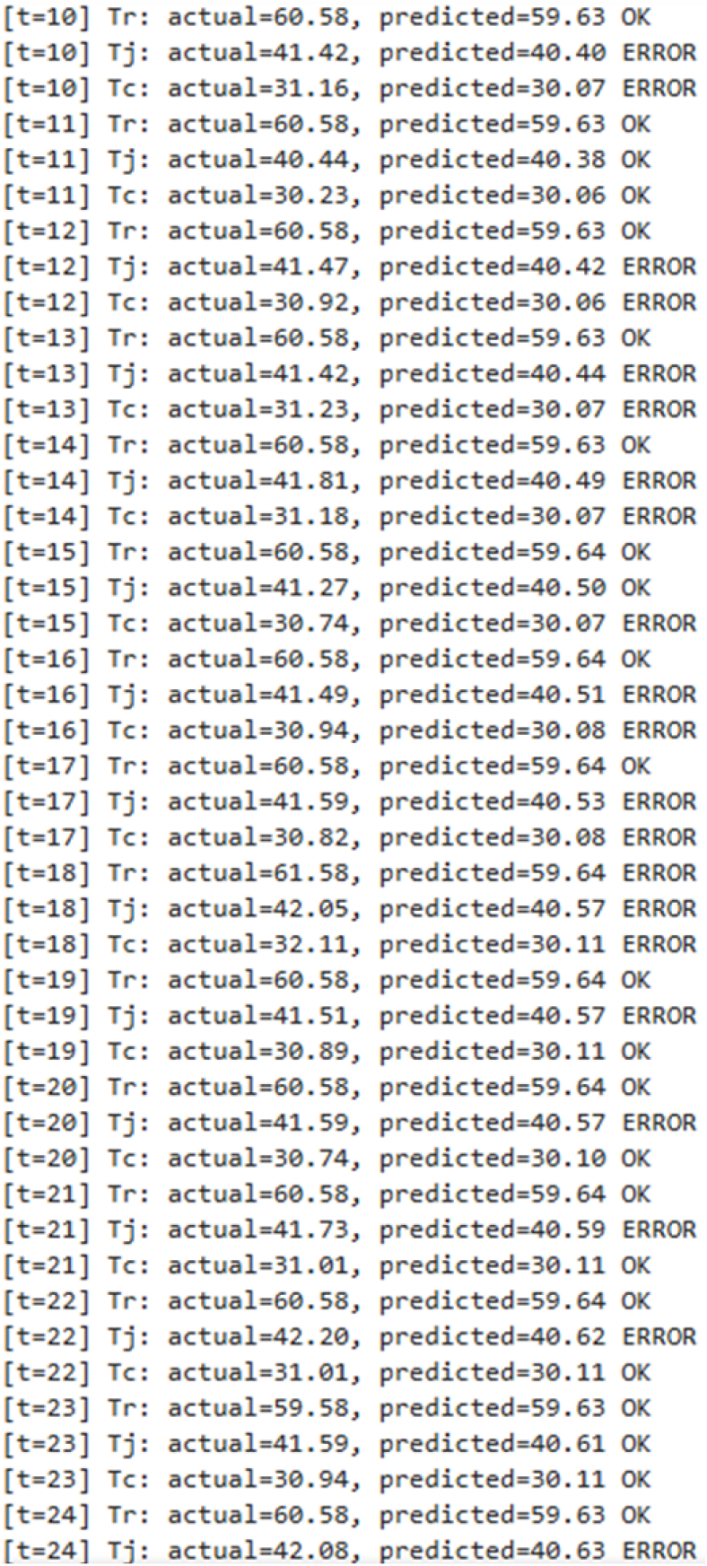
Real-time readings of BR variables of the CS-IMLSTM model.

## 5 Conclusion

This article demonstrates the CS-IMLSTM model-based fault detection method for a BR system. The proposed model was able to detect the introduced faults with high accuracy. The prediction accuracy of the proposed CS-IMLSTM model is 98.6%. When compared with CNN-LSTM accuracy (94.5%) and LSTM accuracy (92.3%), the proposed model significantly outperforms these two methods. This significant improvement in accuracy is achieved mainly due to the adaptation of both temporal feature extraction layers and channel–spatial attention mechanisms for dynamic process monitoring in the proposed model. This model could effectively predict anomalies in the BR temperature variables using real-time data, showing its adaptability for use in industrial control systems for real-time fault identification purposes. The proposed approach can be made scalable by expanding the number of input nodes and extending the time delay. The proposed model was tested in an open-loop configuration on a laboratory-scaled BR system without feedback control. However, this model can be trained to use more data to adapt it for the industrial process fault detection purposes. This model can be extended into a closed-loop model in which the control settings of the reactor can be automatically adjusted using the identified fault to represent the residual dynamics of the system, which enables the approach to be adaptable for hybrid modeling techniques. To develop a hybrid fault diagnosis system, this expansion can be added. This model can be used for reliable and scalable fault detection in chemical processes. This approach can be adapted for next-generation industrial automation and process optimization systems.
